# Making designer mutants in model organisms

**DOI:** 10.1242/dev.102186

**Published:** 2014-11

**Authors:** Ying Peng, Karl J. Clark, Jarryd M. Campbell, Magdalena R. Panetta, Yi Guo, Stephen C. Ekker

**Affiliations:** 1 Department of Biochemistry and Molecular Biology, Mayo Clinic, Rochester, MN 55905, USA; 2 Mayo Addiction Research Center, Mayo Clinic, Rochester, MN 55905, USA; 3 Center for Clinical and Translational Science, Mayo Clinic, Rochester, MN 55905, USA; 4 InSciEd Out and Mayo High School, Rochester Art Center, Mayo Clinic, Rochester, MN 55905, USA; 5 Division of Gastroenterology and Hepatology, Mayo Clinic, Rochester, MN 55905, USA

**Keywords:** Genome engineering, Transcription activator-like effector nuclease (TALEN), Clustered regularly interspaced short palindromic repeats (CRISPR)/CRISPR-associated systems (Cas9), Zinc finger nuclease (ZFN), Model organisms

## Abstract

Recent advances in the targeted modification of complex eukaryotic genomes have unlocked a new era of genome engineering. From the pioneering work using zinc-finger nucleases (ZFNs), to the advent of the versatile and specific TALEN systems, and most recently the highly accessible CRISPR/Cas9 systems, we now possess an unprecedented ability to analyze developmental processes using sophisticated designer genetic tools. In this Review, we summarize the common approaches and applications of these still-evolving tools as they are being used in the most popular model developmental systems. Excitingly, these robust and simple genomic engineering tools also promise to revolutionize developmental studies using less well established experimental organisms.

## Introduction

Modern developmental biology was born out of the fruitful marriage between traditional embryology and genetics. Genetic tools, together with advanced microscopy techniques, serve as the most fundamental means for developmental biologists to elucidate the logistics and the molecular control of growth, differentiation and morphogenesis. For this reason, model organisms with sophisticated and comprehensive genetic tools have been highly favored for developmental studies. Advances made in developmental biology using these genetically amenable models have been well recognized. The Nobel prize in Physiology or Medicine was awarded in 1995 to Edward B. Lewis, Christiane Nüsslein-Volhard and Eric F. Wieschaus for their discoveries on the ‘Genetic control of early structural development’ using *Drosophila melanogaster*, and again in 2002 to John Sulston, Robert Horvitz and Sydney Brenner for their discoveries of ‘Genetic regulation of development and programmed cell death’ using the nematode worm *Caenorhabditis elegans*. These fly and worm systems remain powerful and popular models for invertebrate development studies, while zebrafish (*Danio rerio*), the dual frog species *Xenopus laevis* and *Xenopus tropicalis*, rat (*Rattus norvegicus*), and particularly mouse (*Mus musculus*) represent the most commonly used vertebrate model systems. To date, random or semi-random mutagenesis (‘forward genetic’) approaches have been extraordinarily successful at advancing the use of these model organisms in developmental studies. With the advent of reference genomic data, however, sequence-specific genomic engineering tools (‘reverse genetics’) enable targeted manipulation of the genome and thus allow previously untestable hypotheses of gene function to be addressed.

Homology-directed repair (HDR) is the general approach of using sequence homology for genomic targeting to replace an endogenous locus with foreign DNA and encompasses homologous recombination (HR)-based as well as HR-independent mechanisms (see Box 1). Besides their functional differences in using long double-stranded versus short single-stranded homology sequences, HR-dependent and -independent HDR pathways use overlapping but distinct sets of proteins to fix double-strand breaks (DSBs). In many organisms, isolating desired mutations following random mutagenesis is prohibitively expensive or difficult, and in any case does not enable a particular gene of choice to be disrupted. HDR has therefore become an important tool – particularly in the mouse, where HR is often used to knock out gene function or to generate knock-ins, where a specific sequence can be inserted into the genome at a targeted locus. HR work in mouse embryonic stem (ES) cells has demonstrated the broad utility of HDR-based gene targeting, but this approach has, until recently, been largely restricted to systems where cell culture systems can be used to generate whole organisms.

Box 1. **Common DNA repair pathways**

NHEJ (non-homologous end joining) specifies the DNA repair process where double-strand break (DSB) ends are directly ligated without the need for a homologous template. DNA ends processed via the canonical NHEJ pathway are usually protected from significant 5′-end resection by heterodimeric Ku proteins. Many, but not all, NHEJ-based DNA changes result in small insertions or deletions near the DSB cut site.

HDR (homology-directed repair) is the general category of DNA repair pathways using sequence homology to repair DNA lesions. One common feature of different HDR pathways is the initial 5′-end processing of one or both DNA DSBs. Single-stranded ends generated in this manner are used to search for homologous sequences either from another site in the genome or from a foreign (donor) DNA. HDR may require multiple cellular steps, including DNA replication and other processes. Unlike classical homologous recombination (HR), HDR can use short DNA templates as donors, including single-stranded oligonucleotides. HDR is widely used to replace an endogenous locus with foreign DNA in genome engineering applications. HR is the most well studied mode of homology-directed repair, whereas HDR also includes HR-independent pathways. Canonical HR involves nucleotide sequence exchanges between two similar or identical molecules of DNA. The initiation of efficient homologous recombination requires long stretches of sequence homology between recombining DNAs. HR-independent HDR mechanisms include single-strand annealing (SSA), a process whereby two 5′-processed single-stranded ends are jointed through base-pair complementation, and break-induced replication (BIR), where one 5′-processed DSB uses its homology sequence as template to initiate DNA replication.

Traditional recombinant DNA technology enables molecular biologists to ‘cut and paste’ simpler prokaryotic DNA plasmids with great precision and superb efficiency. However, equivalent applications for studying development in multicellular eukaryotes require novel tools with more stringent sequence specificity and increased versatility, due to higher genomic complexity, as well as a critical efficiency threshold to enable routine applications. Through decades of innovation, promising designer target-specific endonucleases fulfill this important need. In this Review, we will discuss the properties and limitations of different designer endonuclease platforms for the developmental biologist, each of which can be adapted to introduce molecularly distinct site-specific modifications in eukaryotic genomes. We then explore several successful examples of their use. The principles and applications of these designer targeted endonucleases are largely applicable to nearly all genomes tested so far, although with varying efficiencies and limitations. Thus, we will end with a perspective on how these tools can bring a new era of developmental biology: greatly expanding both the depth and scope of potential research avenues, and making this a very stimulating time for the design and execution of diverse experiments to test both long-standing and new hypotheses in the field.

## Designer endonucleases: principle and design

Designer endonuclease-based genome engineering approaches involve introducing a lesion at an intended site of the genome, resulting in a deletion, insertion or replacement of genomic sequence using cellular repair pathways. The key cellular property for genome engineering is the robust recruitment of DNA repair machinery at a desired locus after a double-strand break. The goal of genome engineering is thus to produce reagents, specifically designer endonucleases, that achieve predictable, high-specificity sequence recognition with excellent efficacy. The main inspiration to overcome this hurdle came from understanding how naturally occurring sequence-specific DNA-binding proteins achieve their specificity in reading double-stranded DNA and then using these principles to generate custom enzymes. At present, three major families of designer endonucleases are commonly used: zinc finger nucleases (ZFNs), transcription activator-like effector nucleases (TALENs) and RNA-guided endonucleases.

### Zinc-finger nucleases

**Fig. 1.**
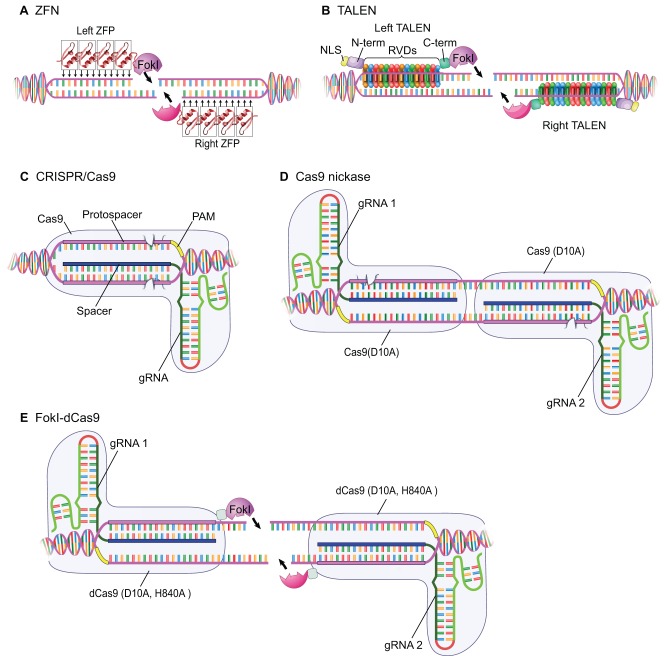
**Custom restriction endonuclease applications.** Schematics summarizing the mechanisms by which the various genome engineering approaches target DNA. (A) Zinc-finger nucleases (ZFNs) recognize DNA using three base pair recognition motifs (ZFPs); fusing several ZFPs in tandem can give unique specificity to a particular genomic locus. The typical system uses two ZFNs recognizing adjacent sequences, each of which is fused to half of the obligate dimer FokI nuclease. (B) Transcription activator-like effector nucleases (TALENs) recognize DNA through modules that include repeat-variable di-residues (RVDs). As with ZFNs, two TALENs are used that cut DNA using the FokI nuclease dimer. In contrast to ZFNs, most recent TALEN backbones include a specific NLS (nuclear localization signal) for better function. (C) CRISPR/Cas9 system recognizes specific DNA using a guide RNA (gRNA)/DNA/Cas9 protein complex based around a tri-nucleotide protospacer adjacent motif (PAM). Two tooth-shaped structures represent Cas9 active sites responsible for DNA cleavage on either stand of dsDNA: the HNH domain cleaves the complementary DNA strand, whereas the RuvC-like domain cleaves the non-complementary DNA strand. (D) Cas9 nickase uses a molecularly modified Cas9(D10A) protein that can only cut on one strand of the recognized gRNA/DNA complex. (E) Nuclease-deficient Cas9/FokI fusion custom restriction endonuclease systems. This approach highly parallels prior work with ZFNs and TALENs, deploying Cas9/gRNA for sequence-specific DNA binding, and the FokI dimer nuclease for locally introducing the double-stranded breaks (DSBs). NLS, nuclear localization sequence; N-term, N terminus; C-term, C terminus. D10A and H840A mutations abolish the Cas9 nuclease activity (dCas9, as 'dead' Cas9).

The Cys2-His2 zinc-finger domain, consisting of ∼30 amino acids in a stereotypical ββα configuration, is one of the most frequently used DNA-binding motifs found in eukaryotic sequence-specific transcriptional factors. Upon binding of a zinc-finger domain to its target site, three base pairs in the major groove of the DNA are in close contact with a few amino acids on the surface of the α-helix. As these contacts mediate the sequence recognition specificity of zinc fingers, modifying the key amino acids on the ‘fingers’ can render a degree of selectivity toward a given three base-pair DNA sequence. Proteins constructed with tandem repeats of engineered, sequence-specific DNA-binding zinc fingers constituted the first successful, custom-designed platform to target DNA sequences (Bibikova et al., 2003; Kim et al., 1996; Porteus and Baltimore, 2003). Fusing such a DNA sequence recognition module with a sequence-independent FokI endonuclease domain produced designer endonucleases that were used to introduce site-specific modifications in the human genome (Urnov et al., 2005). Two individual zinc-finger nucleases (ZFNs) are required to induce a lesion at a single site due to the dimeric requirement for FokI activity (Fig. 1A). To improve sequence specificity, modifications of the dimer interface on the FokI cleavage domain were made to form obligate heterodimers (Doyon et al., 2011). However, a significant drawback for the widespread application of ZFN technology is the limited binding selectivity conferred by the zinc-finger modules, as well as complex context-dependent interactions between adjacent zinc fingers that can alter binding affinity to the DNA (Cornu et al., 2008; Händel et al., 2009). Designing an efficient ZFN is usually not a trivial task, typically involving multiple rounds of tests and modifications. In addition, a lack of consistency, with different design and assembly platforms, makes this technology less accessible to most laboratories (Carroll, 2011; Kim et al., 2010; Ramirez et al., 2008; Sander et al., 2011). Although most genome engineers have switched their strategies to use newer tools (see below), nearly all of this more recent work is based on foundational experiments by pioneering ZFN scientists.

### Transcription activator-like effector nucleases

Transcription activator-like effector (TALE) proteins represent a molecularly unique class of transcription factors from the plant pathogenic bacteria *Xanthomonas* that contain a DNA-binding domain consisting of 33-35 amino-acid modular repeats that each recognize a single DNA base pair (Bogdanove and Voytas, 2011). A simple but stringent correspondence code of DNA recognition was discovered such that two hypervariable amino acids in each TAL domain (repeat-variable di-residues, RVD) can distinguish the four different DNA nucleotides at the recognition site (Boch et al., 2009; Moscou and Bogdanove, 2009). Building on the ZFN work, this new DNA-binding platform facilitated the rapid expansion of DNA-binding proteins using novel TAL DNA-binding domains. TALE nucleases (TALENs) are made by fusing consecutive DNA recognition TAL repeats with the type IIS FokI endonuclease domain to achieve a site-specific DNA lesion (Fig. 1B; Bedell et al., 2012; Christian et al., 2010; Li et al., 2011a; Mahfouz et al., 2011). By the end of 2013, more than 200 reports of independent successful TALEN applications had emerged. From this collected dataset, some conclusions about TALEN applications can be drawn. Current TALEN designs and assembly methodology are extremely effective and can be easily adopted in any lab. Moreover, TALENs have in practice very few restrictions with regard to potential sequence targeting applications, and the use of two 14+ base TALE recognition sites plus a spacer ranging from 13 to 20 bp provides single site recognition potential in even the most complex genomes. Importantly, new synthesis platforms assemble TALENs for as little as US$5 in supply costs per TALEN arm (Liang et al., 2014).

### RNA-guided endonucleases – clustered regularly interspaced short palindromic repeats and their associated systems

Applications using ZFN or TALENs require making target-specific engineering endonuclease constructs involving modular assembly of protein-DNA recognition motifs. Although significant progress has been made to facilitate rapid assembly and cloning (Cermak et al., 2011; Li et al., 2011b; Reyon et al., 2012; Schmid-Burgk et al., 2013), generating a custom engineered protein requires investment and maintenance of plasmid libraries (from 40 to >400 clones), and troubleshooting complex ligations of 6-11 plasmids can be difficult when the reactions are not working well. Recently, an RNA-guided genome engineering system employing components of bacterial adaptive immune response pathway has emerged that does not require custom protein synthesis and instead uses a unique guide RNA (gRNA) along with a single endonuclease protein (Cas9) (Barrangou et al., 2007; Horvath and Barrangou, 2010). Type II clustered regularly interspaced short palindromic repeats and their associated systems (CRISPR/Cas9 systems) from *Streptococcus pyogenes* are the most widely used CRISPR/Cas9 genomic engineering platform to date. Target site recognition relies on the Cas9-mediated Watson-Crick base pairing between a short stretch of CRISPR repeat RNA (originally named as ‘spacer’) and one strand of target DNA (known as the ‘protospacer’ sequence) (Jinek et al., 2012; Makarova et al., 2006). This protospacer sequence must be immediately followed by a ‘NGG’ (or ‘NAG’ with less efficiency) tri-nucleotide protospacer adjacent motif (PAM) on the opposite strand (Mojica et al., 2009); the presence of PAM is crucial for Cas9 target recognition.

To reduce the complexity of the endogenous CRISPR/Cas9 system, a simplified two-component system using a single hybrid hairpin gRNA with a generic tetraloop secondary structure to load Cas9 for sequence-specific DNA cleavage has been developed (Cong et al., 2013; Jinek et al., 2012, 2013; Mali et al., 2013c). Distinct from ZFN and TALEN systems, the separation of the target recognition and nuclease functions on orthogonal components offers extensive flexibility and simplicity for targeted genome manipulations. As Cas9 endonuclease is a universal component for any gRNA-mediated DNA cleavage, transgenic strains expressing Cas9 nuclease endogenously have been made in *Drosophila* (Kondo and Ueda, 2013; Ren et al., 2013; Sebo et al., 2013). Thus, in this simplest CRISPR/Cas9 application method, users need only to design, build and provide one gRNA per targeted lesion in order to introduce genetic changes in those animals.

The CRISPR/Cas9 custom endonucleases are the most accessible custom nuclease system for end users to conduct many site-specific genome manipulations. The gRNA can be *in vitro* transcribed using a designed DNA template (made from synthesized DNA oligonucleotides) or can be supplied in the form of a DNA expression vector, transcribed under the control of a RNA polymerase III promoter (Mali et al., 2013b). Multiplex targeting is also routinely achievable: by providing multiple gRNAs together, lesions can be induced simultaneously at multiple loci (Cong et al., 2013; Wang et al., 2013). Importantly, standard Cas9-mediated custom nuclease applications use a single target sequence for specificity, an approach that is comparable with the specificity found within a single TAL arm and that leads to increased rates of off-target cutting (Mali et al., 2013a). Reducing and/or minimizing the off-target effects of the lower specificity Cas9 system is an active area of genome engineering research (Fig. 1D,E; Ran et al., 2013a; Fu et al., 2014; Guilinger et al., 2014; Tsai et al., 2014).

In contrast to the ZFN and TALEN systems that employ the dimeric type IIS FokI endonuclease and thus function as pairs, the unmodified Cas9 endonuclease monomer has full double-strand DNA cleavage activity once properly guided (Jinek et al., 2012), so only a single gRNA is required. The Cas9 protein contains two independent endonuclease domains homologous to either HNH or RuvC endonucleases (Jinek et al., 2012). Each of these domains cleaves one strand of dsDNA at the target recognition site: the HNH domain cleaves the complementary DNA strand (the strand forming the duplex with gRNA), whereas the RuvC-like domain cleaves the non-complementary DNA strand (Jinek et al., 2012). Recent structural analyses (Nishimasu et al., 2014; Jinek et al., 2014) of CRISPR/Cas9 complexes have revealed a two-lobed structure for Cas9 – a recognition (REC) lobe and a nuclease (NUC) lobe. Cas9 interacts with the RNA-DNA duplex via the REC lobe in a largely sequence-independent manner, implying that the Cas9 protein itself does not confer significant target sequence preference. However, one caveat with the CRISPR/Cas9 system is that gRNA-loaded Cas9 endonuclease cleavage is not completely dependent on linear guide sequence, with some off-target sequences being shown to be cut with similar or even higher efficiency than the designed target site (Cong et al., 2013; Esvelt et al., 2013; Pattanayak et al., 2013; Ran et al., 2013b). In general, mismatch(es) between the first 12 nucleotides (nt) of the gRNA and the DNA target are not well tolerated, suggesting high sequence specificity in the PAM-proximal region. However, mismatches beyond the first 12 nt can be compatible with efficient cleavage (Cong et al., 2013). A recent biophysical study (Sternberg et al., 2014) on the thermodynamic properties of Cas9 binding has provided a likely explanation for the features of specificity outlined above. Although this general rule regarding specificity holds true, it is an over-simplification, and the sequence recognition specificity of the CRISPR system is a topic of active investigation (Cho et al., 2014; Esvelt et al., 2013; Pattanayak et al., 2013; Ran et al., 2013b; Ren et al., 2013). Notably, shorter gRNAs with up to 5000-fold reduction in off-target effects have been recently described (Fu et al., 2014). Adding two additional G nucleotides on the 5′ end of gRNA in some instances improves the specificity of the CRISPR/Cas9 system (Cho et al., 2014), possibly by altering gRNA stability, concentration or secondary structure. The relaxation of sequence specificity of the RNA-guided endonuclease system remains the biggest challenge so far for its use in genome engineering (Jinek et al., 2014; Sternberg et al., 2014).

To reduce this problem of potential DNA lesions at off-target sites, two promising practical solutions have been developed to impose a requirement for two gRNA target recognition sites. The first uses a Cas9 mutant with only a single-strand endonuclease activity such as the D10A mutant that cleaves only the strand complementary with the gRNA (Cho et al., 2014; Esvelt et al., 2013; Ran et al., 2013a). Such a mutant Cas9 endonuclease can generate only a single-stranded DNA lesion and is thus named a Cas9 ‘nickase’ (Jinek et al., 2012). When two single-stranded lesions are introduced simultaneously on opposite strands, via a Cas9 nickase guided by independent gRNAs recognizing adjacent target sequences, a combined double-stranded break will be made at the targeted site (Fig. 1D). The most recent addition is the use of two nuclease-deficient Cas9 proteins fused to the FokI nuclease domain, deployed in a way that is almost analogous to ZFNs and TALENs (Fig. 1E; Guilinger et al., 2014; Tsai et al., 2014). In both of these systems, the specificity is inherently higher than with one gRNA (Cho et al., 2014; Esvelt et al., 2013; Ran et al., 2013a). The increase of targeting fidelity with both approaches comes with some sacrifice in efficiency: both Cas9 nickase and FokI-dCAS9 demonstrate lower endonuclease activity than wild-type Cas9. In addition, a key limitation of this approach is the requirement for two closely spaced PAM sequences in the same target region of the genome (Fig. 1D,E).

Beyond the type II CRISPR/Cas9 system from *Streptococcus pyogenes* that is currently in popular use today, other similar type II CRISPR/Cas9 systems are also being developed (Chylinski et al., 2013; Esvelt et al., 2013; Fonfara et al., 2013). The major differences between these diverse CRISPR systems lie in the PAM sequence recognized by the endonuclease. This provides additional flexibility in identifying suitable sites for targeted modifications, with the goal of relieving the requirement for an NGG sequence.

### Practical considerations using designer endonucleases: specificity, efficiency and target site flexibility

Achieving high targeting specificity is important for targetable nuclease applications. One reason for the relatively low frequency of off-target cleavage by ZFNs and TALENs is the dimeric requirement for FokI DNA cleavage. The extent of potential off-target lesions using dimeric ZFN and TALEN systems has been investigated and supports the broad conclusion of limited background effects (Gabriel et al., 2011; Gupta et al., 2011; Hockemeyer et al., 2011; Mussolino et al., 2011; Osborn et al., 2013; Pattanayak et al., 2011; Tesson et al., 2011). In the standard CRISPR-Cas9 system, the reduced sequence recognition stringency that is observed might come from the evolutionary advantage of an adaptive acquired immune system: a less stringent Cas9 endonuclease that tolerates some mismatches between the crRNA and an evolving invading genome may have been selectively preserved through evolution (Carroll, 2013). The Cas9/FokI systems (Fig. 1E) are currently the best alternative to the standard Cas9 nuclease when stringent target selection is desired (Cho et al., 2014; Esvelt et al., 2013; Ran et al., 2013a). Regardless of the choice of designer endonuclease, under situations where the delivery dose reaches saturation, a clear nuclease-associated cellular toxicity can be observed; keeping the nuclease activity at the lowest practical level would be beneficial to restrict DNA lesions on the targeted locus and reduce off-target effects (Cho et al., 2014; Ran et al., 2013b).

For model organisms, the effects of off-target lesions can be reduced through breeding to untargeted animals: unlinked mutations tend to be diluted quickly within the genetic pool through generational passing. For example, the zebrafish genome is encoded by 25 pairs of chromosomes; chromosomal segregation through out-crossing eliminates unintended changes on 49/50 linkage groups. In model organisms with much fewer chromosomes (such as *Drosophila* with four chromosome pairs), meiotic recombination in the germline also helps to dilute off-target lesions, thus only loci near the target sequence are likely to be refractory to genetic dilution. Modern designer nucleases have proven particularly useful in model systems in which homozygosity cannot be rapidly achieved through breeding. In these systems, efficient biallelic chromosome conversion is desirable, and both CRISPRs and TALENs have been shown to accelerate the production of homozygosity in human pluripotent stem cells (González et al., 2014), as well as in other cell culture systems, with biallelic conversion rates up to 39% (Tan et al., 2013). Given the potential for off-target lesions, it is in general good practice to include proper controls to ensure the generated molecular allele is responsible for the documented phenotype. Importantly, and similar to traditional means of mutagenesis, genetic mapping of a mutant phenotype to a specific lesion locus does not prove causality. Obtaining additional independent data, such as another mutant allele mapped to the same gene, knockdown phenocopy (morpholinos and/or RNAi) and ultimately phenotypic rescue using the wild-type gene or gene product will be necessary to consolidate the causal relationship. The ability to make specific DNA lesions in the genome at ease with these tools should not overshadow their intrinsic limitations, as it is virtually impossible to exclude the possibility of additional lesions introduced and retained in the genome.

The proliferation of these new designer nucleases has occurred over a very short period of time, and they have yet to be developed into truly foolproof tools that offer consistently high targeting efficiency with tight specificity in every system. Thus, it is still possible that a designer nuclease made by following the current ‘best’ guidelines may work poorly (Cong et al., 2013; Mali et al., 2013b; Ran et al., 2013b). It is particularly puzzling for some CRISPR/Cas9 experiments that targeting efficiency achieved by different gRNAs may vary by a few orders of magnitude, even if the Cas9 endonuclease is supplied endogenously at a constant level (Kondo and Ueda, 2013; Sebo et al., 2013). Systematic efforts are needed to pinpoint the major factors that contribute to the variations in targeting efficiencies and to provide further guidelines for improvement. One such factor that is likely to be especially confounding for *in vitro* and somatic cell work is the epigenetic state, particularly the DNA methylation status, of the target genomic sequence. Some TALENs work less efficiently on methylated DNA (Dupuy et al., 2013), while limited reports suggest the Cas9 system is less sensitive to DNA methylation status (Ran et al., 2013b). Such differences in DNA methylation sensitivity are likely due to their different modes of target recognition: the 5-methyl group on C does not affect proper base pairing with gRNA, but might abolish the protein:DNA interaction in the major groove for ZFNs and TALENs. It is, however, noteworthy that CpG methylation seems to be negatively correlated with Cas9 binding to DNA *in vivo* (Wu et al., 2014). Such seemingly contradictory observations could be resolved by the strong indication that chromatin accessibility is a major determinant of Cas9 binding to DNA *in vivo* (Kuscu et al., 2014; Wu et al., 2014), but that, once bound, the endonuclease activity of Cas9 is unaffected by DNA methylation status.

Among the more popular tools, target site flexibility is a major practical difference between TALENs and CRISPRs. TALENs provide high precision and the greatest options for target site selection with current tools, with no absolute sequence requirements, though the presence of a 5′ T base in each TALEN arm often provides the highest activity (reviewed by Campbell et al., 2013; Lamb et al., 2013; Tsuji et al., 2013). Standard CRISPR systems require only a single PAM sequence in the targeted genomic sequence (Fig. 1C), but some CRISPR systems also have sequence requirements due to the RNA expression system used to make the gRNAs (Hwang et al., 2013); sequence constraints are therefore greater than for TALENs. The use of dual gRNA-based CRISPR/Cas9 systems (Fig. 1D,E) will increase the targeting specificity while reducing options for target site selection.

## Applications: designer genome modifications

**Fig. 2.**
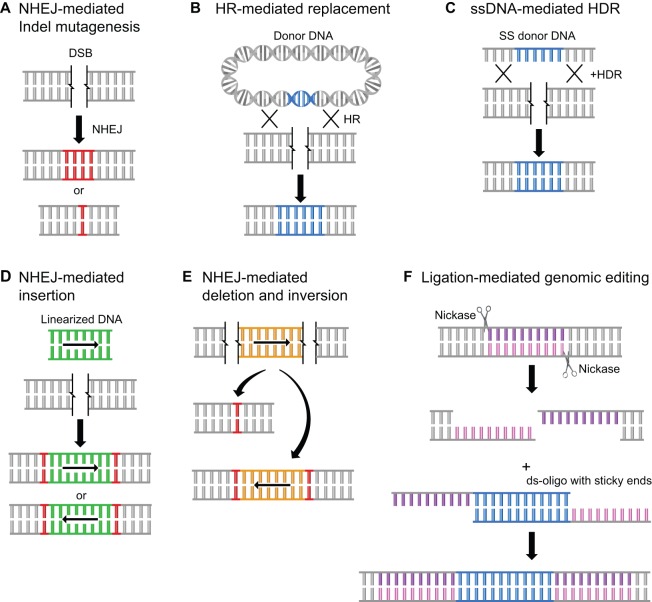
**Classes of genomic changes made possible by readily available custom restriction endonucleases.** (A) Indel mutagenesis examples (red bases) due to error-prone non-homologous end-joining (NHEJ)-mediated DNA repair at the double-stranded break (DSB). (B) Homologous recombination (HR)-mediated DNA replacement (blue bases) using a dsDNA donor. (C) Single-stranded DNA oligonucleotide (ssDNA)-mediated homology-directed repair (HDR; green bases). (D) NHEJ-mediated insertion of DNA (green bases represent introduced foreign sequence, whereas red bases reflect potential changes from error-prone NHEJ-based repair). (E) NHEJ-mediated deletion and genomic inversion (yellow bases show DNA that may be inverted or deleted). (F) Ligation-mediated genomic editing (blue bases represent insertion; purple bases of different shades represent local duplications on complementary DNA strands).

**Fig. 3.**
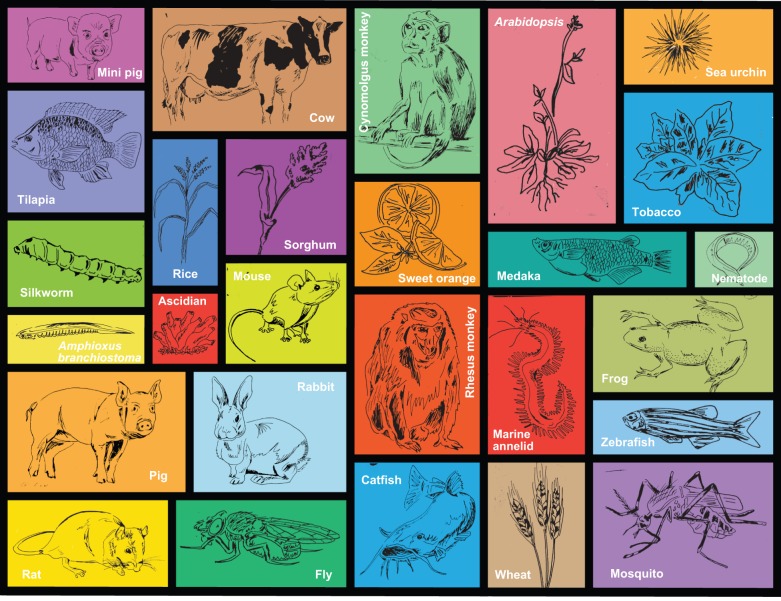
**A snapshot of the landscape of genomic engineering in multicellular organisms.** The advent of new designer nucleases has revolutionized the world of targeted biological genetic modifications, including both conventional model systems, such as mouse and fruit fly, as well as a veritable menagerie of plants and animals. This diversity enables hypotheses – old and new – to be genetically tested in the world of developmental biology. Here, we depict a snapshot showing the wide range of multicellular organisms with successful applications of genome engineering tools to date: miniature swine (Ossabaw swine; Carlson et al., 2012), bovids (Carlson et al., 2012), cynomolgus monkey (*Macaca fascicularis*; Liu et al., 2014a; Liu et al., 2014b; Niu et al., 2014), *Arabidopsis thaliana* (Christian et al., 2013; Jiang et al., 2013), sea urchin (Hosoi et al., 2014), tobacco (Zhang et al., 2013), tilapia (Li et al., 2013), rice (Shan et al., 2013; Jiang et al., 2013; Xie and Yang, 2013), sorghum (Jiang et al., 2013), sweet (Jia and Wang, 2014), medaka (Ansai et al., 2013), nematode (Wood et al., 2011), silkworm (*Bombyx mori*; Daimon et al., 2014; Ma et al., 2012), amphioxus (*Branchiostoma belcheri*; Li et al., 2014), the ascidian *Ciona intestinalis* (Yoshida et al., 2014; Treen et al., 2014), mouse, pig (Tan et al., 2013), rabbit (Tang et al., 2014), Rhesus monkey (Liu et al., 2014a), the marine annelid *Platynereis dumerilii* (Bannister et al., 2014), frog (Blitz et al., 2013; Guo et al., 2014), zebrafish, rat (Geurts et al., 2009), fruit fly (*Drosophila melanogaster*), catfish (Dong et al., 2014), wheat (Upadhyay et al., 2013) and mosquito (*Aedes aegypti*; Aryan et al., 2013; Smidler et al., 2013).

The designer nucleases discussed above achieve only the first step of genome engineering applications: the introduction of a double-strand break at a specified genomic site. These lesions are corrected by cellular DNA repair machineries, during which genomic DNA might be modified at the targeted site. In general, DSBs are commonly fixed either through error-prone non-homologous end-joining (NHEJ) (joining two distal DNA breakpoints; see Box 1 and Fig. 2A) or homology-directed repair (HDR) (which requires the presence of a donor DNA with homology to the sequences distal to DNA break point; Fig. 2B,C). NHEJ involves only the two DNA sequences flanking the DSB and is a very efficient repair outcome in many cell types. For this reason, NHEJ-based local sequence modification has been the most widely used genome engineering application that has been successfully demonstrated in almost all organisms tested so far (Fig. 3; and see below). HDR is necessarily a more-complex reaction as it involves the DNA sequences adjacent to the DSB in addition to the added complexity of a third DNA molecule that serves as the donor template (see Box 1). Consequently, HDR is much less efficient than NHEJ in most systems.

The outcome of these repair processes can be unpredictable, but they can also lead to a range of useful mutagenic lesions for genome engineering applications, including deletion or inversion of endogenous sequences, or insertion of exogenous sequences (discussed further below). As we have little manipulative capability to guide the cellular DNA repair process to achieve a given outcome, current best practice is to screen through the pool of targeted cells or organisms for a specific modification at the targeted locus. Indeed, the diversity of outcomes can be exploited in model systems, as a range of results can be clonally amplified by subsequent germline propagation. Given the high degree of conservation of DSB repair pathways in eukaryotes, the diverse genomic engineering applications are translatable between different model organisms, although it should be noted that different DNA repair pathways can be differentially favored between different cell types, even within the same organism. In the following sections, we present an overview of the main types of genome editing that are commonly being achieved via different mechanisms of DSB repair induced by custom endonucleases (see also Table 1 for a summary of key examples). Although we provide examples of a few model organisms in which a particular application has been demonstrated so far, we encourage readers to go beyond the established examples and explore the most effective approach with their favorite system.

**Table 1.**
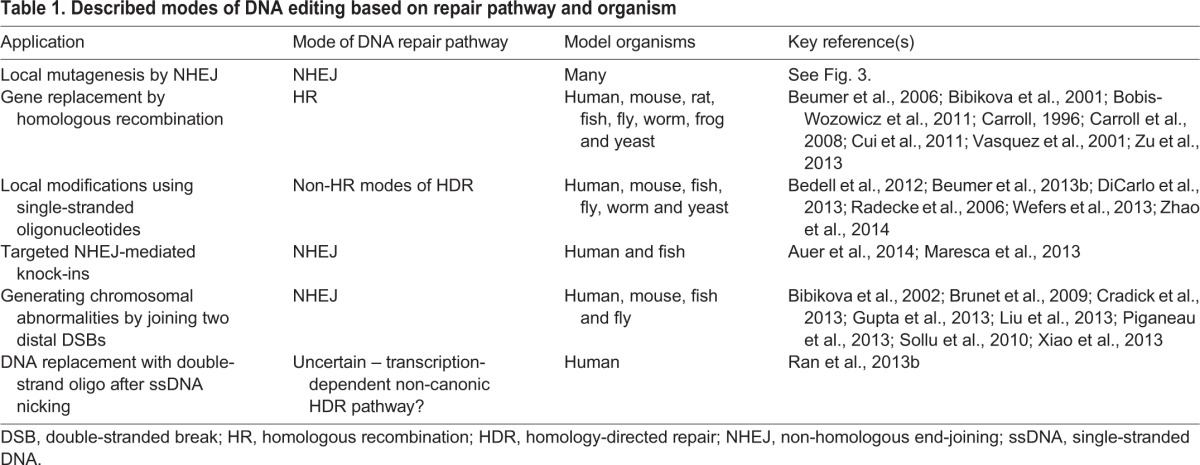
**Described modes of DNA editing based on repair pathway and organism**

### Mutagenesis by NHEJ and detection methodology

The most straightforward application of designer nucleases is to induce a small mutation at a particular site within the genome (Fig. 2A). If such a site is within a protein open reading frame, DSB repair by the error-prone NHEJ pathway can induce a deleterious missense or nonsense mutation in the protein of interest. Missense mutants generated in this manner can be used as a quick means to study structure-function relationships of a few key amino acid residues *in vivo*. Nonsense or frame-shift mutations will likely make truncated proteins or no protein at all, which can be valuable tools for genetic analysis. The efficiency of DSB generation by the latest generation of engineered nucleases can be sufficiently high that both alleles of the same site are modified within an individual organism *in vivo* (see, for example, Bedell et al., 2012; Beumer et al., 2013a; Tan et al., 2013).

Molecular assays are typically used to detect insertion or deletion (indel) mutations generated by designer nucleases. The local DNA sequence change may be reflected by RFLP (restriction fragment length polymorphism) if a restriction enzyme site is included around the target sequence. A more general approach takes advantage of local DNA sequence changes that lead to alterations in melting temperature of a PCR-based DNA fragment. Thus, HRMA (high resolution melting analysis) (Wittwer, 2009) or WAVE (based on high resolution temperature-modulated high-pressure liquid chromatography) (Yu et al., 2006) can be used to detect mutations after PCR amplification of the targeted sequence. Alternatively, a mixture of near-identical DNA with local sequence variations (created via indel generation) will make annealed duplexes during PCR with mismatched single-stranded bases at the mutated site. Such single-stranded ‘bubbles’ in the PCR product can be recognized and cut by Cel1 or T7E1 nucleases, generating two shorter DNA fragments. A sensitive nuclease assay is thus used widely for mutation detection (Qiu et al., 2004). The newest addition to the toolkit is the recent development of single-molecule real-time (SMRT) DNA-sequencing technique, which can measure in parallel the frequency of different editing events at any given targeted locus within a large DNA population (Hendel et al., 2014).

### Improving homologous recombination efficiency by DSBs introduced on the recipient DNA

Homologous recombination (HR) was the preferred methodology for generating site-specific modifications before the widespread use of designer nucleases. A problem with traditional HR has been low efficiency, thus limiting the model systems where it is feasible. For example, classical HR-mediated gene targeting in *Drosophila* is so inefficient that the donor DNA cannot be directly provided as plasmids for embryonic injection due to the limitation in numbers of injections one can practically handle (Rong and Golic, 2000). However, introducing a targeted DSB results in a much-improved HR efficiency (Fig. 2B). Pioneering work with ZFNs demonstrated that the HR rate following a DSB improved up to 100-fold (Beumer et al., 2008; Bibikova et al., 2001; Porteus and Baltimore, 2003). Recent trials with TALENs and CRISPR systems have also shown a significant improvement of HR efficiency (Baena-Lopez et al., 2013; Beumer et al., 2013a). Thus, in flies, introducing DSBs onto the target site by various nuclease systems has enabled the direct injection of DNA donors into fly embryos as a viable and faster alternative to the traditional transgenic method used to generate modified flies by homologous recombination (Baena-Lopez et al., 2013; Gratz et al., 2014). The functional advantage of designer nucleases to enhance the rate of HR-mediated gene replacement has been successfully demonstrated in all major model organisms (including yeast, worm, fly, fish and mammals; see Table 1).

### Making small indels or modifications by homology-directed repair using single-stranded oligonucleotides

Alternatively, homology-directed repair can be used to introduce local modifications at DNA lesion sites (Fig. 2C). A short homology arm of only 10-30 nt is usually sufficient to induce HDR-mediated DNA repair of lesions introduced by targeted endonucleases *in vivo* (Bedell et al., 2012; see Campbell et al., 2013 for a review). Such minimal homology requirement enables synthesized single-stranded DNA (ssDNA) oligonucleotides to serve as a suitable template to repair DSBs via a still poorly defined HDR pathway. This approach is particularly applicable for engineering small changes, including as little as a single nucleotide. Interestingly, one molecular signature that distinguishes this HDR pathway from traditional homologous recombination is the asymmetry of many modified loci around the DSB. Using ssDNA as donor and reference, the 3′ side tends to be repaired accurately, whereas small indels that are more characteristic of NHEJ-based repair are often found at the 5′ side; this has been seen in mammalian tissue culture cells, and zebrafish and mouse embryos (Orlando et al., 2010; Bedell et al., 2012; Wefers et al., 2013). This molecular asymmetry around an otherwise symmetrical DSB suggests that mechanisms involving DNA synthesis may play an important role in this process.

Besides the homology arms on its 5′ and 3′ ends matching the sequences of boundary sequences at the DNA DSB, the donor ssDNA oligonucleotide can incorporate virtually any sequence in the middle as long as the overall length of oligonucleotide is within the reasonable range of synthesis (currently, up to ∼200 bases for routine DNA oligonucleotide synthesis). Thus, small DNA elements can be introduced at a particular position in the selected locus. This could result in, for example, the insertion of a short defined stretch of amino acids into a particular site of a protein, an epitope tag fusing in frame with the targeted protein for endogenous tagging, or a recombination site enabling further site-specific engineering. Owing to the ease of making donor DNA through chemical synthesis of short oligonucleotides, such methods for creating local sequence modifications have been successfully demonstrated in many organisms (including yeast, worm, fly, fish and mammals; see Table 1).

In human cell lines, precise deletions have been achieved using an appropriately designed repair ssDNA (Chen et al., 2011). In some mammalian cells, the efficiency of resection is low, thus limiting the application of this strategy to generating small deletions close to the lesion site (Yang et al., 2013). Alternatively, larger deletions can be generated by inducing two distinct DSBs, which can be repaired using an ssDNA donor harboring homology ends to DNA sequences flanking the distal sides of each cut (Cong et al., 2013; Ran et al., 2013a). Overall, this strategy can be used to generate deletions, to manipulate the sequence of a particular stretch of the targeted protein or to introduce a DNA element at a particular site for further site-specific manipulations. The successful use of ssDNA donor-mediated HDR repair also applies in cases using double Cas9 nickases, specifically when a 30-70 nt 5′ overhang is generated (Ran et al., 2013a).

### Generating targeted NHEJ-mediated knock-ins

The ability to make site-specific DNA lesions creates new avenues for site-specific transgenesis. Traditionally, site-specific transgenesis either relies on inefficient HR-mediated knock-in or previously deposited recombination sites (such as attP or loxP). A highly efficient site-specific transgenesis method without such limitations would be greatly beneficial to developmental biologists.

Two recent reports on high-efficiency site-specific gene insertions indicate that such an approach can be routinely adopted with the assistance of designer endonucleases (Maresca et al., 2013; Auer et al., 2014). Both reports exploit the property of NHEJ to fuse DSBs regardless of their sequence (Fig. 2D): chromosomal lesions were previously noted to frequently ‘absorb’ external DNA fragments (Gabriel et al., 2011; Lin and Waldman, 2001a,b; Miller et al., 2004). A strategy named ‘ObLiGaRe’ (obligate ligation-gated recombination) has been developed in mammalian cells (Maresca et al., 2013), whereby a ZFN or TALEN pair would cut simultaneously at one site in the genomic DNA and a similar site in the donor plasmid. The NHEJ-mediated repair could then ‘ligate’ the plasmid into the genomic site through the DSBs. Upon insertion of the ligated product, the transgene insertion will abolish the TALEN recognition site, thus enabling stable site-specific transgenesis. Plasmids up to 15 kb have been integrated through this method, whereas no off-targeted insertion was observed.

Similarly, an *in vivo* study using zebrafish confirmed the specificity and effectiveness of designer nuclease mediated site-specific transgenesis (Auer et al., 2014). When a ‘bait’ sequence was incorporated in a donor plasmid, simultaneous cutting of the genomic DNA and the plasmid using CRISPR/Cas9 system enabled high efficiency site-specific transgenesis. Importantly, multiple gRNAs can be used simultaneously, so the targets on the genomic DNA and donor plasmid can be distinct (Jinek et al., 2012). This finding also supports the notion that site-specific insertion of foreign DNA is indeed through homology-independent end joining, which can happen in either orientation with error-prone junctions. In theory, any linearized dsDNA could be incorporated into the lesion site, although initial tests using linearized plasmids were inefficient, perhaps due to toxicity in fish embryos (Auer et al., 2014). To date, these novel approaches have come from vertebrate models (mouse and human cells, as well as zebrafish); however, the prevailing mode of NHEJ DSB repair suggests similar or related approaches will also prove effective for invertebrate systems.

### Generating deficiencies by joining two distal DSBs

Chromosomal deficiencies, duplication and inversions are traditionally induced with random mutagenesis tools such as DNA-damaging gamma rays. Chromosomal abnormalities with precise boundaries can be important tools to map developmental regulatory landscapes, and can be achieved with high efficiency and versatility using designer nucleases (Liu et al., 2013; Piganeau et al., 2013; Xiao et al., 2013). Successful examples of this application have been at least demonstrated for mammals, fish and flies. By design, genomic segments from tens of kilobases up to 15 megabases can be deleted by the combination of two pairs of custom nucleases, including TALENs, Cas9 nucleases or the derived nickases (Cong et al., 2013; Fujii et al., 2013; Ran et al., 2013a). Although chromosomal translocations have been observed as an unfavorable by-product of CRISPR-induced lesions due to off-target lesions on different chromosomes (Cradick et al., 2013), these observations also suggest two distal DNA fragments without homology can be efficiently joined via the NHEJ pathway, and was recently used to model genomic rearrangements observed in human cancer (Choi and Meyerson, 2014). Although the deletions are generally very reproducible in nature, these chromosomal abnormalities are ultimately repaired by the error-prone NHEJ repair pathway. As a result, the local sequence around the junctions of the two distal DNA segments is highly variable at the individual nucleotide level between independent deletion mutants. Such variations need to be taken into consideration for downstream biological studies using such deficiencies.

### DNA replacement with double-strand (ds) oligo after ssDNA nicking

The ability of a DNA nickase (such as Cas9 nickase or equivalent ZFN or TALEN nickases) (Kim et al., 2012; Wu et al., 2014) to generate a lesion on a particular strand of DNA at the target site enables the generation of 5′ or 3′ single-stranded DNA overhangs when two nickases make lesions at adjacent sites (Fig. 2F). These sticky ends with defined sequences can be used to incorporate foreign dsDNA fragments with compatible sticky ends. This is conceptually very similar to sticky end-mediated molecular cloning after restriction enzyme digestion in recombinant DNA technology. Even though this application has not been extensively tested beyond one pilot study in human cells, where a 148 bp dsDNA oligo was successfully inserted via its 5′ overhangs (Ran et al., 2013a), such an approach could in theory circumvent some practical restrictions of HDR-mediated insertion of ssDNA (size limitation of insertion and the dependence of homology overhang of significant size).

## Conclusions

The availability of a wide range of designer nuclease tools with successful applications in model organisms is revolutionizing the way basic developmental processes can be studied. For example, making static knockout alleles using NHEJ is now standard practice in most developmental biology systems, something either difficult or impossible to do just a few years ago. Moreover, the arrival of versatile, robust and simple genomic engineering tools means that genetic analysis of developmental processes should no longer be confined to traditional model organisms, but can be extended to less well-established organisms, where genetic tools have been lagging behind. Fig. 3 shows a snapshot of the published systems to date. Examples of organisms in which these tools have been successfully applied range from commercially important livestock (e.g. cows and pigs; Carlson et al., 2012), to species important for disease research (e.g. mosquito; Aryan et al., 2013) and from systems that have previously been difficult to target genetically (e.g. *Xenopus*; Blitz et al., 2013; Guo et al., 2014), to newer models for evo-devo studies (e.g. *Ciona*; Treen et al., 2014). These tools are broadly applicable for NHEJ-induced mutations and more sophisticated approaches are likely to succeed in many cases. One caveat is that the design of the targeted nucleases relies on high-quality genomic sequence, so ongoing genomic sequencing and assembly efforts for such models will need to continue. This will not only provide a clear genetic blueprint of their development and physiology, but will offer immediate access to many of these new genetic manipulation tools. Another bottleneck in some organisms will be the development of methods for introducing nucleic acids encoding designer nucleases and their repair substrates into developing embryos and germline cells. With the advent of these new technologies in diverse species, comparative and functional studies between developmentally divergent and evolutionarily related species need no longer be simply tested in the species with the accessible genetic tools, but will likely be cross-examined in any pair-wise or even multiplexed phylogenetic combinations. Bringing models that were previously inaccessible to genetic manipulation into functional developmental studies will thus equip every developmental biologist with a novel evolutionary prospective.

Beyond the ready generation of static alleles, these tools offer new options for precise and sophisticated molecular genetic manipulations. Engineering the genomic locus of interest at its endogenous site has many advantages. For example, with single base resolution of genomic editing, it is feasible to mutate specifically a crucial amino acid or to influence the choice of multiple alternative splicing events in order to study the function of a particular protein isoform, all conducted within the context of a complete genomic complex with regulatory and other control elements intact. Introducing an epitope tag at a chosen position in a protein should also be straighforward, thus circumventing the need to generate a protein-specific antibody for immunolocalization or immunoprecipitation. It would even be conceivable to epitope tag every transcription factor within the genomes of key model organisms for systematic chromatin immunoprecipitation approaches. In addition to manipulations of protein-coding regions in the genome, developmental biologists are now equipped with a feasible means with which to probe the vast regulatory genomic landscape (which remains largely unexplored by traditional random mutagenesis approach) and to study the gene regulation network within an intact genomic context. Many developmentally regulated genes possess multiple regulatory elements, which work together to produce complex spatial and temporal expression patterns during development. Approaches using genomic engineering to examine their roles within their normal genomic context will be crucial to understand the interplay between distinct regulatory elements in controlling gene expression.

Furthermore, the ease of targeted manipulation of any genomic region enables the next era beyond genetics in developmental studies, by offering avenues to monitor and manipulate the transcription or epigenetic status of individual loci (Maeder et al., 2013a; Mendenhall et al., 2013) with single cell precision in relation to pattern formation and cell fate determination. Such designer transcriptional and epigenetic regulators (Table 2) build on similar platforms as various designer nucleases that we discussed previously. A significant current drawback shared among such approaches is that multiple targeting modules are usually required to act synergistically on a single locus to bring about any biological meaningful perturbation. Yet further refinement of this class of targeting regulators will definitely hold even greater promise to the new generation of developmental biologists.

**Table 2.**
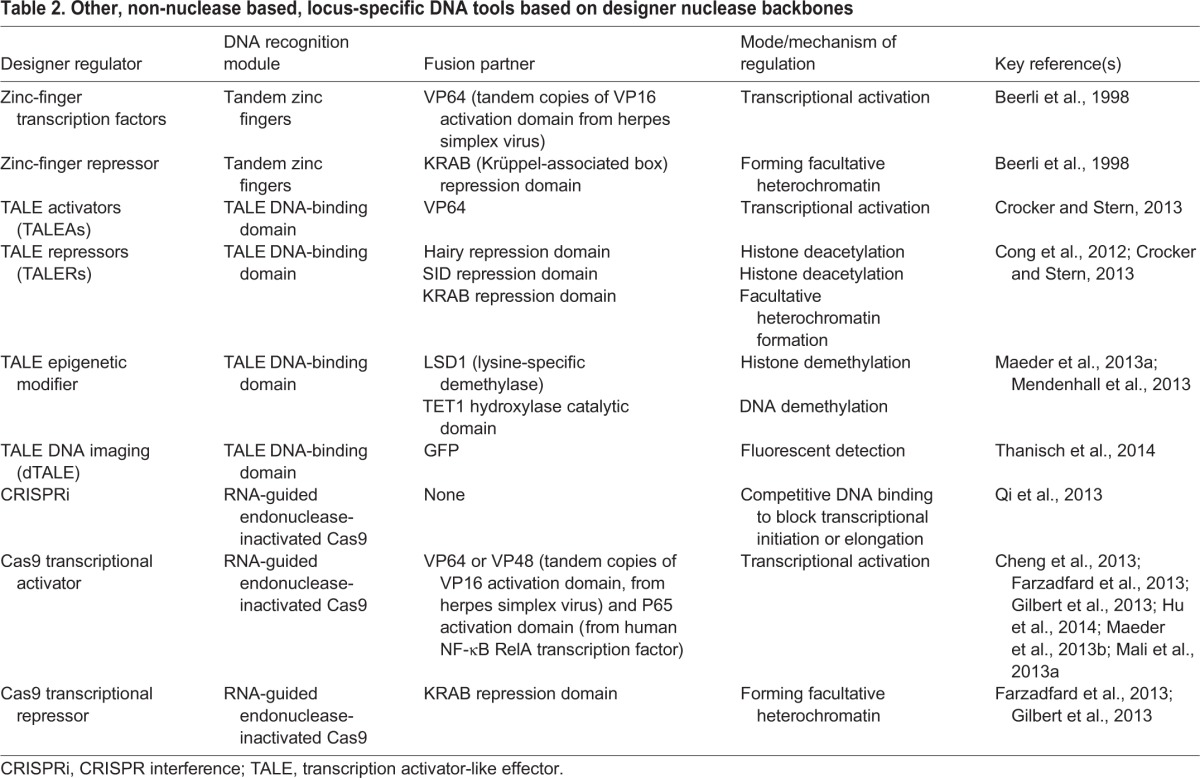
**Other, non-nuclease based, locus-specific DNA tools based on designer nuclease backbones**

## References

[DEV102186C1] Ansai S., Sakuma T., Yamamoto T., Ariga H., Uemura N., Takahashi R. and Kinoshita M. (2013). Efficient targeted mutagenesis in medaka using custom-designed transcription activator-like effector nucleases. *Genetics* 193, 739-749 10.1534/genetics.112.147645 23288935 PMC3583995

[DEV102186C2] Aryan A., Anderson M. A. E., Myles K. M. and Adelman Z. N. (2013). TALEN-based gene disruption in the dengue vector Aedes aegypti. *PLoS ONE* 8, e60082 10.1371/journal.pone.0060082 23555893 PMC3605403

[DEV102186C3] Auer T. O., Duroure K., De Cian A., Concordet J.-P. and Del Bene F. (2014). Highly efficient CRISPR/Cas9-mediated knock-in in zebrafish by homology-independent DNA repair. *Genome Res.* 24, 142-153 10.1101/gr.161638.113 24179142 PMC3875856

[DEV102186C4] Baena-Lopez L. A., Alexandre C., Mitchell A., Pasakarnis L. and Vincent J.-P. (2013). Accelerated homologous recombination and subsequent genome modification in Drosophila. *Development* 140, 4818-4825 10.1242/dev.100933 24154526 PMC3833436

[DEV102186C5] Bannister S., Antonova O., Polo A., Lohs C., Hallay N., Valinciute A., Raible F. and Tessmar-Raible K. (2014). TALENs mediate efficient and heritable mutation of endogenous genes in the marine annelid platynereis dumerilii. *Genetics* 197, 77-89 10.1534/genetics.113.161091 24653002 PMC4012502

[DEV102186C6] Barrangou R., Fremaux C., Deveau H., Richards M., Boyaval P., Moineau S., Romero D. A. and Horvath P. (2007). CRISPR provides acquired resistance against viruses in prokaryotes. *Science* 315, 1709-1712 10.1126/science.1138140 17379808

[DEV102186C7] Bedell V. M., Wang Y., Campbell J. M., Poshusta T. L., Starker C. G., Krug R. G. II, Tan W., Penheiter S. G., Ma A. C., Leung A. Y. H. et al. (2012). In vivo genome editing using a high-efficiency TALEN system. *Nature* 491, 114-118 10.1038/nature11537 23000899 PMC3491146

[DEV102186C8] Beerli R. R., Segal D. J., Dreier B. and Barbas C. F. 3rd (1998). Toward controlling gene expression at will: specific regulation of the erbB-2/HER-2 promoter by using polydactyl zinc finger proteins constructed from modular building blocks. *Proc. Natl Acad. Sci. USA* 95, 14628-14633 10.1073/pnas.95.25.14628 9843940 PMC24500

[DEV102186C9] Beumer K., Bhattacharyya G., Bibikova M., Trautman J. K. and Carroll D. (2006). Efficient gene targeting in Drosophila with zinc-finger nucleases. *Genetics* 172, 2391-2403 10.1534/genetics.105.052829 16452139 PMC1456366

[DEV102186C10] Beumer K. J., Trautman J. K., Bozas A., Liu J.-L., Rutter J., Gall J. G. and Carroll D. (2008). Efficient gene targeting in Drosophila by direct embryo injection with zinc-finger nucleases. *Proc. Natl. Acad. Sci. USA* 105, 19821-19826 10.1073/pnas.0810475105 19064913 PMC2604940

[DEV102186C11] Beumer K. J., Trautman J. K., Christian M., Dahlem T. J., Lake C. M., Hawley R. S., Grunwald D. J., Voytas D. F. and Carroll D. (2013a). Comparing zinc finger nucleases and transcription activator-like effector nucleases for gene targeting in Drosophila. *G3 (Bethesda)* 3, 1717-1725 10.1534/g3.113.007260 23979928 PMC3789796

[DEV102186C12] Beumer K. J., Trautman J. K., Mukherjee K. and Carroll D. (2013b). Donor DNA utilization during gene targeting with Zinc-finger nucleases. *G3 (Bethesda)* 3, 657-664. 10.1534/g3.112.005439 PMC3618352 23550125

[DEV102186C13] Bibikova M., Carroll D., Segal D. J., Trautman J. K., Smith J., Kim Y.-G. and Chandrasegaran S. (2001). Stimulation of homologous recombination through targeted cleavage by chimeric nucleases. *Mol. Cell. Biol.* 21, 289-297 10.1128/MCB.21.1.289-297.2001 11113203 PMC88802

[DEV102186C14] Bibikova M., Golic M., Golic K. G. and Carroll D. (2002). Targeted chromosomal cleavage and mutagenesis in Drosophila using zinc-finger nucleases. *Genetics* 161, 1169-1175. 12136019 10.1093/genetics/161.3.1169 PMC1462166

[DEV102186C15] Bibikova M., Beumer K., Trautman J. K. and Carroll D. (2003). Enhancing gene targeting with designed zinc finger nucleases. *Science* 300, 764 10.1126/science.1079512 12730594

[DEV102186C16] Blitz I. L., Biesinger J., Xie X. and Cho K. W. Y. (2013). Biallelic genome modification in F(0) Xenopus tropicalis embryos using the CRISPR/Cas system. *Genesis* 51, 827-834 10.1002/dvg.22719 24123579 PMC4039559

[DEV102186C17] Bobis-Wozowicz S., Osiak A., Rahman S. H. and Cathomen T. (2011). Targeted genome editing in pluripotent stem cells using zinc-finger nucleases. *Methods* 53, 339-346 10.1016/j.ymeth.2010.12.019 21185378

[DEV102186C146] Boch J., Scholze H., Schornack S., Landgraf A., Hahn S., Kay S., Lahaye T., Nickstadt A. and Bonas U. (2009). Breaking the code of DNA binding specificity of TAL-type III effectors. *Science* 326, 1509-1512 10.1126/science.1178811 19933107

[DEV102186C18] Bogdanove A. J. and Voytas D. F. (2011). TAL effectors: customizable proteins for DNA targeting. *Science* 333, 1843-1846 10.1126/science.1204094 21960622

[DEV102186C19] Brunet E., Simsek D., Tomishima M., DeKelver R., Choi V. M., Gregory P., Urnov F., Weinstock D. M. and Jasin M. (2009). Chromosomal translocations induced at specified loci in human stem cells. *Proc. Natl. Acad. Sci. USA* 106, 10620-10625 10.1073/pnas.0902076106 19549848 PMC2700748

[DEV102186C20] Campbell J. M., Hartjes K. A., Nelson T. J., Xu X. and Ekker S. C. (2013). New and TALENted genome engineering toolbox. *Circ. Res.* 113, 571-587 10.1161/CIRCRESAHA.113.301765 23948583 PMC3965580

[DEV102186C21] Carlson D. F., Tan W., Lillico S. G., Stverakova D., Proudfoot C., Christian M., Voytas D. F., Long C. R., Whitelaw C. B. A. and Fahrenkrug S. C. (2012). Efficient TALEN-mediated gene knockout in livestock. *Proc. Natl. Acad. Sci. USA* 109, 17382-17387 10.1073/pnas.1211446109 23027955 PMC3491456

[DEV102186C22] Carroll D. (1996). Homologous genetic recombination in Xenopus: mechanism and implications for gene manipulation. *Prog. Nucleic Acid Res. Mol. Biol.* 54, 101-125 10.1016/S0079-6603(08)60361-X 8768073

[DEV102186C23] Carroll D. (2011). Genome engineering with zinc-finger nucleases. *Genetics* 188, 773-782 10.1534/genetics.111.131433 21828278 PMC3176093

[DEV102186C24] Carroll D (2013). Staying on target with CRISPR-Cas. *Nat Biotechnol* 31, 807-809 10.1038/nbt.2684 24022156

[DEV102186C25] Carroll D., Beumer K. J., Morton J. J., Bozas A. and Trautman J. K. (2008). Gene targeting in Drosophila and Caenorhabditis elegans with zinc-finger nucleases. *Methods Mol. Biol.* 435, 63-77 10.1007/978-1-59745-232-8_5 18370068

[DEV102186C26] Cermak T., Doyle E. L., Christian M., Wang L., Zhang Y., Schmidt C., Baller J. A., Somia N. V., Bogdanove A. J. and Voytas D. F. (2011). Efficient design and assembly of custom TALEN and other TAL effector-based constructs for DNA targeting. *Nucleic Acids Res.* 39, e82 10.1093/nar/gkr218 21493687 PMC3130291

[DEV102186C27] Chen F., Pruett-Miller S. M., Huang Y., Gjoka M., Duda K., Taunton J., Collingwood T. N., Frodin M. and Davis G. D. (2011). High-frequency genome editing using ssDNA oligonucleotides with zinc-finger nucleases. *Nat. Methods* 8, 753-755 10.1038/nmeth.1653 21765410 PMC3617923

[DEV102186C28] Cheng A. W., Wang H., Yang H., Shi L., Katz Y., Theunissen T. W., Rangarajan S., Shivalila C. S., Dadon D. B. and Jaenisch R (2013). Multiplexed activation of endogenous genes by CRISPR-on, an RNA-guided transcriptional activator system. *Cell Res.* 23, 1163-1171 10.1038/cr.2013.122 23979020 PMC3790238

[DEV102186C29] Cho S. W., Kim S., Kim Y., Kweon J., Kim H. S., Bae S. and Kim J.-S. (2014). Analysis of off-target effects of CRISPR/Cas-derived RNA-guided endonucleases and nickases. *Genome Res.* 24, 132-141 10.1101/gr.162339.113 24253446 PMC3875854

[DEV102186C30] Choi P. S. and Meyerson M. (2014). Targeted genomic rearrangements using CRISPR/Cas technology. *Nat. Commun.* 5, 3728 10.1038/ncomms4728 24759083 PMC4170920

[DEV102186C31] Christian M., Cermak T., Doyle E. L., Schmidt C., Zhang F., Hummel A., Bogdanove A. J. and Voytas D. F. (2010). Targeting DNA double-strand breaks with TAL effector nucleases. *Genetics* 186, 757-761 10.1534/genetics.110.120717 20660643 PMC2942870

[DEV102186C32] Chylinski K., Le Rhun A. and Charpentier E. (2013). The tracrRNA and Cas9 families of type II CRISPR-Cas immunity systems. *RNA Biol.* 10, 726-737 10.4161/rna.24321 23563642 PMC3737331

[DEV102186C33] Cong L., Zhou R., Kuo Y. C., Cunniff M. and Zhang F (2012). Comprehensive interrogation of natural TALE DNA-binding modules and transcriptional repressor domains. *Nature Commun.* 3, 968. 10.1038/ncomms1962 PMC3556390 22828628

[DEV102186C34] Cong L., Ran F. A., Cox D., Lin S., Barretto R., Habib N., Hsu P. D., Wu X., Jiang W., Marraffini L. A. et al. (2013). Multiplex genome engineering using CRISPR/Cas systems. *Science* 339, 819-823 10.1126/science.1231143 23287718 PMC3795411

[DEV102186C35] Cornu T. I., Thibodeau-Beganny S., Guhl E., Alwin S., Eichtinger M., Joung J. K. and Cathomen T. (2008). DNA-binding specificity is a major determinant of the activity and toxicity of zinc-finger nucleases. *Mol. Ther.* 16, 352-358 10.1038/sj.mt.6300357 28178540

[DEV102186C36] Cradick T. J., Fine E. J., Antico C. J. and Bao G. (2013). CRISPR/Cas9 systems targeting beta-globin and CCR5 genes have substantial off-target activity. *Nucleic Acids Res.* 41, 9584-9592 10.1093/nar/gkt714 23939622 PMC3814385

[DEV102186C37] Crocker J. and Stern D. L. (2013). TALE-mediated modulation of transcriptional enhancers in vivo. *Nat Methods* 10, 762-767 10.1038/nmeth.2543 23817068 PMC3733453

[DEV102186C38] Cui X., Ji D., Fisher D. A., Wu Y., Briner D. M. and Weinstein E. J. (2011). Targeted integration in rat and mouse embryos with zinc-finger nucleases. *Nat. Biotechnol.* 29, 64-67 10.1038/nbt.1731 21151125

[DEV102186C39] Daimon T., Kiuchi T. and Takasu Y. (2014). Recent progress in genome engineering techniques in the silkworm, Bombyx mori. *Dev. Growth Differ.* 56, 14-25 10.1111/dgd.12096 24175911

[DEV102186C40] DiCarlo J. E., Norville J. E., Mali P., Rios X., Aach J. and Church G. M. (2013). Genome engineering in Saccharomyces cerevisiae using CRISPR-Cas systems. *Nucleic Acids Res.* 41, 4336-4343 10.1093/nar/gkt135 23460208 PMC3627607

[DEV102186C41] Dong Z., Ge J., Xu Z., Dong X., Cao S., Pan J. and Zhao Q. (2014). Generation of myostatin B knockout yellow catfish (Tachysurus fulvidraco) using transcription activator-like effector nucleases. *Zebrafish* 11, 265-274 10.1089/zeb.2014.0974 24813227 PMC4050710

[DEV102186C42] Doyon Y., Vo T. D., Mendel M. C., Greenberg S. G., Wang J., Xia D. F., Miller J. C., Urnov F. D., Gregory P. D. and Holmes M. C. (2011). Enhancing zinc-finger-nuclease activity with improved obligate heterodimeric architectures. *Nat. Methods* 8, 74-79 10.1038/nmeth.1539 21131970

[DEV102186C43] Esvelt K. M., Mali P., Braff J. L., Moosburner M., Yaung S. J. and Church G. M. (2013). Orthogonal Cas9 proteins for RNA-guided gene regulation and editing. *Nat. Methods* 10, 1116-1121 10.1038/nmeth.2681 24076762 PMC3844869

[DEV102186C44] Farzadfard F., Perli S. D. and Lu T. K. (2013). Tunable and multifunctional eukaryotic transcription factors based on CRISPR/Cas. *ACS Synthetic Biol.* 2, 604-613 10.1021/sb400081r PMC3805333 23977949

[DEV102186C45] Fonfara I., Le Rhun A., Chylinski K., Makarova K. S., Lecrivain A. L., Bzdrenga J., Koonin E. V. and Charpentier E. (2013). Phylogeny of Cas9 determines functional exchangeability of dual-RNA and Cas9 among orthologous type II CRISPR-Cas systems. *Nucleic Acids Res.* 42, 2577-2590 10.1093/nar/gkt1074 24270795 PMC3936727

[DEV102186C46] Fu Y., Sander J. D., Reyon D., Cascio V. M. and Joung J. K. (2014). Improving CRISPR-Cas nuclease specificity using truncated guide RNAs. *Nat. Biotechnol.* 32, 279-284 10.1038/nbt.2808 24463574 PMC3988262

[DEV102186C47] Fujii W., Kawasaki K., Sugiura K. and Naito K. (2013). Efficient generation of large-scale genome-modified mice using gRNA and CAS9 endonuclease. *Nucleic Acids Res.* 41, e187 10.1093/nar/gkt772 23997119 PMC3814358

[DEV102186C48] Gabriel R., Lombardo A., Arens A., Miller J. C., Genovese P., Kaeppel C., Nowrouzi A., Bartholomae C. C., Wang J., Friedman G. et al. (2011). An unbiased genome-wide analysis of zinc-finger nuclease specificity. *Nat. Biotechnol.* 29, 816-823 10.1038/nbt.1948 21822255

[DEV102186C49] Geurts A. M., Cost G. J., Freyvert Y., Zeitler B., Miller J. C., Choi V. M., Jenkins S. S., Wood A., Cui X., Meng X. et al. (2009). Knockout rats via embryo microinjection of zinc-finger nucleases. *Science* 325, 433. 10.1126/science.1172447 PMC2831805 19628861

[DEV102186C50] Gilbert L. A., Larson M. H., Morsut L., Liu Z., Brar G. A., Torres S. E., Stern-Ginossar N., Brandman O., Whitehead E. H., Doudna J. A. et al. (2013). CRISPR-mediated modular RNA-guided regulation of transcription in eukaryotes. *Cell* 154, 442-451 10.1016/j.cell.2013.06.044 23849981 PMC3770145

[DEV102186C51] González F., Zhu Z., Shi Z.-D., Lelli K., Verma N., Li Q. V. and Huangfu D. (2014). An iCRISPR platform for rapid, multiplexable, and inducible genome editing in human pluripotent stem cells. *Cell Stem Cell* 15, 215-226 10.1016/j.stem.2014.05.018 24931489 PMC4127112

[DEV102186C155] Gratz S. J., Ukken F. P., Rubinstein C. D., Thiede G., Donohue L. K., Cummings A. M., and O'Connor-Giles K. M. (2014). Highly specific and efficient CRISPR/Cas9-catalyzed homology-directed repair in *Drosophila*. *Genetics* 196, 961-971 10.1534/genetics.113.160713 24478335 PMC3982687

[DEV102186C52] Guilinger J. P., Thompson D. B. and Liu D. R. (2014). Fusion of catalytically inactive Cas9 to FokI nuclease improves the specificity of genome modification. *Nat. Biotechnol.* 32, 577-582 10.1038/nbt.2909 24770324 PMC4263420

[DEV102186C53] Guo X., Zhang T., Hu Z., Zhang Y., Shi Z., Wang Q., Cui Y., Wang F., Zhao H. and Chen Y. (2014). Efficient RNA/Cas9-mediated genome editing in Xenopus tropicalis. *Development* 141, 707-714 10.1242/dev.099853 24401372

[DEV102186C54] Gupta A., Meng X., Zhu L. J., Lawson N. D. and Wolfe S. A. (2011). Zinc finger protein-dependent and -independent contributions to the in vivo off-target activity of zinc finger nucleases. *Nucleic Acids Res.* 39, 381-392 10.1093/nar/gkq787 20843781 PMC3017618

[DEV102186C55] Gupta A., Hall V. L., Kok F. O., Shin M., McNulty J. C., Lawson N. D. and Wolfe S. A. (2013). Targeted chromosomal deletions and inversions in zebrafish. *Genome Res.* 23, 1008-1017 10.1101/gr.154070.112 23478401 PMC3668355

[DEV102186C56] Händel E.-M., Alwin S. and Cathomen T. (2009). Expanding or restricting the target site repertoire of zinc-finger nucleases: the inter-domain linker as a major determinant of target site selectivity. *Mol. Ther.* 17, 104-111 10.1038/mt.2008.233 19002164 PMC2834978

[DEV102186C57] Hendel A., Kildebeck E. J., Fine E. J., Clark J. T., Punjya N., Sebastiano V., Bao G. and Porteus M. H. (2014). Quantifying genome-editing outcomes at endogenous loci with SMRT sequencing. *Cell Rep.* 7, 293-305 10.1016/j.celrep.2014.02.040 24685129 PMC4015468

[DEV102186C58] Hockemeyer D., Wang H., Kiani S., Lai C. S., Gao Q., Cassady J. P., Cost G. J., Zhang L., Santiago Y., Miller J. C. et al. (2011). Genetic engineering of human pluripotent cells using TALE nucleases. *Nat. Biotechnol.* 29, 731-734 10.1038/nbt.1927 21738127 PMC3152587

[DEV102186C59] Horvath P. and Barrangou R. (2010). CRISPR/Cas, the immune system of bacteria and archaea. *Science* 327, 167-170 10.1126/science.1179555 20056882

[DEV102186C60] Hosoi S., Sakuma T., Sakamoto N. and Yamamoto T. (2014). Targeted mutagenesis in sea urchin embryos using TALENs. *Dev. Growth Differ.* 56, 92-97 10.1111/dgd.12099 24262038

[DEV102186C61] Hu J., Lei Y., Wong W. K., Liu S., Lee K. C., He X., You W., Zhou R., Guo J. T., Chen X. et al (2014). Direct activation of human and mouse Oct4 genes using engineered TALE and Cas9 transcription factors. *Nucleic Acids Res.* 42, 4375-4390 10.1093/nar/gku109 24500196 PMC3985678

[DEV102186C62] Hwang W. Y., Fu Y., Reyon D., Maeder M. L., Tsai S. Q., Sander J. D., Peterson R. T., Yeh J. R. and Joung J. K. (2013). Efficient genome editing in zebrafish using a CRISPR-Cas system. *Nat. Biotechnol.* 31, 227-229 10.1038/nbt.2501 23360964 PMC3686313

[DEV102186C63] Jia H. and Wang N. (2014). Targeted genome editing of sweet orange using Cas9/sgRNA. *PLoS One* 9, e93806 10.1371/journal.pone.0093806 24710347 PMC3977896

[DEV102186C64] Jiang W., Zhou H., Bi H., Fromm M., Yang B. and Weeks D. P. (2013). Demonstration of CRISPR/Cas9/sgRNA-mediated targeted gene modification in Arabidopsis, tobacco, sorghum and rice. *Nucleic Acids Res.* 41, e188 10.1093/nar/gkt780 23999092 PMC3814374

[DEV102186C65] Jinek M., Chylinski K., Fonfara I., Hauer M., Doudna J. A. and Charpentier E. (2012). A programmable dual-RNA-guided DNA endonuclease in adaptive bacterial immunity. *Science* 337, 816-821 10.1126/science.1225829 22745249 PMC6286148

[DEV102186C66] Jinek M., East A., Cheng A., Lin S., Ma E. and Doudna J. (2013). RNA-programmed genome editing in human cells. *Elife* 2, e00471 10.7554/eLife.00471 23386978 PMC3557905

[DEV102186C67] Jinek M., Jiang F., Taylor D. W., Sternberg S. H., Kaya E., Ma E., Anders C., Hauer M., Zhou K., Lin S. et al. (2014). Structures of Cas9 endonucleases reveal RNA-mediated conformational activation. *Science* 343, 1247997 10.1126/science.1247997 24505130 PMC4184034

[DEV102186C68] Kim Y. G., Cha J. and Chandrasegaran S. (1996). Hybrid restriction enzymes: zinc finger fusions to Fok I cleavage domain. *Proc. Natl. Acad. Sci. USA* 93, 1156-1160 10.1073/pnas.93.3.1156 8577732 PMC40048

[DEV102186C69] Kim J.-S., Lee H. J. and Carroll D. (2010). Genome editing with modularly assembled zinc-finger nucleases. *Nat. Methods* 7, 91; author reply 91–92 10.1038/nmeth0210-91a 20111032 PMC2987589

[DEV102186C70] Kim E., Kim S., Kim D. H., Choi B.-S., Choi I.-Y. and Kim J.-S. (2012). Precision genome engineering with programmable DNA-nicking enzymes. *Genome Res.* 22, 1327-1333 10.1101/gr.138792.112 22522391 PMC3396373

[DEV102186C71] Kondo S. and Ueda R. (2013). Highly improved gene targeting by germline-specific Cas9 expression in Drosophila. *Genetics* 195, 715-721 10.1534/genetics.113.156737 24002648 PMC3813859

[DEV102186C72] Kuscu C., Arslan S., Singh R., Thorpe J. and Adli M. (2014). Genome-wide analysis reveals characteristics of off-target sites bound by the Cas9 endonuclease. *Nat. Biotechnol.* 32, 677-683 10.1038/nbt.2916 24837660

[DEV102186C73] Lamb B. M., Mercer A. C. and Barbas C. F. 3rd. (2013). Directed evolution of the TALE N-terminal domain for recognition of all 5' bases. *Nucleic Acids Res.* 41, 9779-9785 10.1093/nar/gkt754 23980031 PMC3834825

[DEV102186C74] Li T., Huang S., Jiang W. Z., Wright D., Spalding M. H., Weeks D. P. and Yang B. (2011a). TAL nucleases (TALNs): hybrid proteins composed of TAL effectors and FokI DNA-cleavage domain. *Nucleic Acids Res.* 39, 359-372 10.1093/nar/gkq704 20699274 PMC3017587

[DEV102186C75] Li T., Huang S., Zhao X., Wright D. A., Carpenter S., Spalding M. H., Weeks D. P. and Yang B. (2011b). Modularly assembled designer TAL effector nucleases for targeted gene knockout and gene replacement in eukaryotes. *Nucleic Acids Res.* 39, 6315-6325 10.1093/nar/gkr188 21459844 PMC3152341

[DEV102186C76] Li M. H., Yang H. H., Li M. R., Sun Y. L., Jiang X. L., Xie Q. P., Wang T. R., Shi H. J., Sun L. N., Zhou L. Y. et al (2013). Antagonistic roles of Dmrt1 and Foxl2 in sex differentiation via estrogen production in tilapia as demonstrated by TALENs. *Endocrinology* 154, 4814-4825 10.1210/en.2013-1451 24105480

[DEV102186C77] Li G., Feng J., Lei Y., Wang J., Wang H., Shang L. K., Liu D. T., Zhao H., Zhu Y. and Wang Y. Q. (2014). Mutagenesis at specific genomic loci of amphioxus Branchiostoma belcheri using TALEN method. *J. Genet. Genom.* 41, 215-219 10.1016/j.jgg.2014.02.003 PMC4535448 24780619

[DEV102186C78] Liang J., Chao R., Abil Z., Bao Z. and Zhao H. (2014). FairyTALE: a high-throughput TAL effector synthesis platform. *ACS Synthetic Biol.* 3, 67-73 10.1021/sb400109p 24237314

[DEV102186C79] Lin Y. and Waldman A. S. (2001a). Capture of DNA sequences at double-strand breaks in mammalian chromosomes. *Genetics* 158, 1665-1674. 11514454 10.1093/genetics/158.4.1665 PMC1461771

[DEV102186C80] Lin Y. and Waldman A. S. (2001b). Promiscuous patching of broken chromosomes in mammalian cells with extrachromosomal DNA. *Nucleic Acids Res.* 29, 3975-3981. 11574679 10.1093/nar/29.19.3975 PMC60240

[DEV102186C81] Liu Y., Luo D., Zhao H., Zhu Z., Hu W. and Cheng C. H. K. (2013). Inheritable and precise large genomic deletions of non-coding RNA genes in zebrafish using TALENs. *PLoS ONE* 8, e76387 10.1371/journal.pone.0076387 24130773 PMC3794983

[DEV102186C82] Liu Y., Luo D., Zhao H., Zhu Z., Hu W. and Cheng C. H. (2013). Inheritable and precise large genomic deletions of non-coding RNA genes in zebrafish using TALENs. *PLoS One* 8, e76387 10.1371/journal.pone.0076387 24130773 PMC3794983

[DEV102186C83] Liu H., Chen Y., Niu Y., Zhang K., Kang Y., Ge W., Liu X., Zhao E., Wang C., Lin S. et al (2014a). TALEN-mediated gene mutagenesis in rhesus and cynomolgus monkeys. *Cell Stem Cell* **14**, 323-328 10.1016/j.stem.2014.01.018 PMC4024384 24529597

[DEV102186C84] Liu Z., Zhou X., Zhu Y., Chen Z. F., Yu B., Wang Y., Zhang C. C., Nie Y. H., Sang X., Cai Y. J. et al (2014b). Generation of a monkey with MECP2 mutations by TALEN-based gene targeting. *Neurosci. Bull.* 30, 381-386 10.1007/s12264-014-1434-8 24838303 PMC5562616

[DEV102186C85] Ma S., Zhang S., Wang F., Liu Y., Liu Y., Xu H., Liu C., Lin Y., Zhao P. and Xia Q. (2012). Highly efficient and specific genome editing in silkworm using custom TALENs. *PLoS One* 7, e45035 10.1371/journal.pone.0045035 23028749 PMC3445556

[DEV102186C86] Maeder M. L., Angstman J. F., Richardson M. E., Linder S. J., Cascio V. M., Tsai S. Q., Ho Q. H., Sander J. D., Reyon D., Bernstein B. E. et al (2013a). Targeted DNA demethylation and activation of endogenous genes using programmable TALE-TET1 fusion proteins. *Nat. Biotechnol.* 31, 1137-1142 10.1038/nbt.2726 24108092 PMC3858462

[DEV102186C87] Maeder M. L., Linder S. J., Cascio V. M., Fu Y., Ho Q. H. and Joung J. K. (2013b). CRISPR RNA-guided activation of endogenous human genes. *Nat. Methods* 10, 977-979 10.1038/nmeth.2598 23892898 PMC3794058

[DEV102186C88] Mahfouz M. M., Li L., Shamimuzzaman M., Wibowo A., Fang X. and Zhu J.-K. (2011). De novo-engineered transcription activator-like effector (TALE) hybrid nuclease with novel DNA binding specificity creates double-strand breaks. *Proc. Natl. Acad. Sci. USA* 108, 2623-2628 10.1073/pnas.1019533108 21262818 PMC3038751

[DEV102186C89] Makarova K. S., Grishin N. V., Shabalina S. A., Wolf Y. I. and Koonin E. V. (2006). A putative RNA-interference-based immune system in prokaryotes: computational analysis of the predicted enzymatic machinery, functional analogies with eukaryotic RNAi, and hypothetical mechanisms of action. *Biol. Direct* 1, 7 10.1186/1745-6150-1-7 16545108 PMC1462988

[DEV102186C90] Mali P., Esvelt K. M. and Church G. M. (2013a). Cas9 as a versatile tool for engineering biology. *Nat. Methods* 10, 957-963 10.1038/nmeth.2649 24076990 PMC4051438

[DEV102186C91] Mali P., Yang L., Esvelt K. M., Aach J., Guell M., DiCarlo J. E., Norville J. E. and Church G. M. (2013b). RNA-guided human genome engineering via Cas9. *Science* 339, 823-826 10.1126/science.1232033 23287722 PMC3712628

[DEV102186C92] Mali P., Yang L., Esvelt K. M., Aach J., Guell M., DiCarlo J. E., Norville J. E. and Church G. M. (2013c). RNA-guided human genome engineering via Cas9. *Science* 339, 823-826 10.1126/science.1232033 23287722 PMC3712628

[DEV102186C93] Maresca M., Lin V. G., Guo N. and Yang Y. (2013). Obligate ligation-gated recombination (ObLiGaRe): custom-designed nuclease-mediated targeted integration through nonhomologous end joining. *Genome Res.* 23, 539-546 10.1101/gr.145441.112 23152450 PMC3589542

[DEV102186C94] Mendenhall E. M., Williamson K. E., Reyon D., Zou J. Y., Ram O., Joung J. K. and Bernstein B. E. (2013). Locus-specific editing of histone modifications at endogenous enhancers. *Nat. Biotechnol.* 31, 1133-1136 10.1038/nbt.2701 24013198 PMC3858395

[DEV102186C95] Miller D. G., Petek L. M. and Russell D. W. (2004). Adeno-associated virus vectors integrate at chromosome breakage sites. *Nat. Genet.* 36, 767-773 10.1038/ng1380 15208627

[DEV102186C96] Mojica F. J. M., Diez-Villasenor C., Garcia-Martinez J. and Almendros C. (2009). Short motif sequences determine the targets of the prokaryotic CRISPR defence system. *Microbiology* 155, 733-740 10.1099/mic.0.023960-0 19246744

[DEV102186C97] Moscou M. J. and Bogdanove A. J. (2009). A simple cipher governs DNA recognition by TAL effectors. *Science* 326, 1501 10.1126/science.1178817 19933106

[DEV102186C98] Mussolino C., Morbitzer R., Lutge F., Dannemann N., Lahaye T. and Cathomen T. (2011). A novel TALE nuclease scaffold enables high genome editing activity in combination with low toxicity. *Nucleic Acids Res.* 39, 9283-9293 10.1093/nar/gkr597 21813459 PMC3241638

[DEV102186C99] Nishimasu H., Ran F. A., Hsu P. D., Konermann S., Shehata S. I., Dohmae N., Ishitani R., Zhang F. and Nureki O. (2014). Crystal structure of cas9 in complex with guide RNA and target DNA. *Cell* 156, 935-949 10.1016/j.cell.2014.02.001 24529477 PMC4139937

[DEV102186C100] Niu Y., Shen B., Cui Y., Chen Y., Wang J., Wang L., Kang Y., Zhao X., Si W., Li W. et al (2014). Generation of gene-modified cynomolgus monkey via Cas9/RNA-mediated gene targeting in one-cell embryos. *Cell* 156, 836-843 10.1016/j.cell.2014.01.027 24486104

[DEV102186C101] Orlando S. J., Santiago Y., DeKelver R. C., Freyvert Y., Boydston E. A., Moehle E. A., Choi V. M., Gopalan S. M., Lou J. F., Li J. et al (2010). Zinc-finger nuclease-driven targeted integration into mammalian genomes using donors with limited chromosomal homology. *Nucleic Acids Res.* 38, e152 10.1093/nar/gkq512 20530528 PMC2926620

[DEV102186C102] Osborn M. J., Starker C. G., McElroy A. N., Webber B. R., Riddle M. J., Xia L., DeFeo A. P., Gabriel R., Schmidt M., von Kalle C. et al. (2013). TALEN-based gene correction for epidermolysis bullosa. *Mol. Ther.* 21, 1151-1159 10.1038/mt.2013.56 23546300 PMC3677309

[DEV102186C103] Pattanayak V., Ramirez C. L., Joung J. K. and Liu D. R. (2011). Revealing off-target cleavage specificities of zinc-finger nucleases by in vitro selection. *Nat. Methods* 8, 765-770 10.1038/nmeth.1670 21822273 PMC3164905

[DEV102186C104] Pattanayak V., Lin S., Guilinger J. P., Ma E., Doudna J. A. and Liu D. R. (2013). High-throughput profiling of off-target DNA cleavage reveals RNA-programmed Cas9 nuclease specificity. *Nat. Biotechnol.* 31, 839-843 10.1038/nbt.2673 23934178 PMC3782611

[DEV102186C105] Piganeau M., Ghezraoui H., De Cian A., Guittat L., Tomishima M., Perrouault L., Rene O., Katibah G. E., Zhang L., Holmes M. C. et al. (2013). Cancer translocations in human cells induced by zinc finger and TALE nucleases. *Genome Res.* 23, 1182-1193 10.1101/gr.147314.112 23568838 PMC3698511

[DEV102186C106] Porteus M. H. and Baltimore D. (2003). Chimeric nucleases stimulate gene targeting in human cells. *Science* 300, 763 10.1126/science.1078395 12730593

[DEV102186C107] Qi L. S., Larson M. H., Gilbert L. A., Doudna J. A., Weissman J. S., Arkin A. P. and Lim W. A. (2013). Repurposing CRISPR as an RNA-guided platform for sequence-specific control of gene expression. *Cell* 152, 1173-1183 10.1016/j.cell.2013.02.022 23452860 PMC3664290

[DEV102186C108] Qiu P., Shandilya H., D'Alessio J. M., O'Connor K., Durocher J. and Gerard G. F. (2004). Mutation detection using Surveyor nuclease. *Biotechniques* 36, 702-707. 15088388 10.2144/04364PF01

[DEV102186C109] Radecke F., Peter I., Radecke S., Gellhaus K., Schwarz K. and Cathomen T. (2006). Targeted chromosomal gene modification in human cells by single-stranded oligodeoxynucleotides in the presence of a DNA double-strand break. *Mol. Ther.* 14, 798-808 10.1016/j.ymthe.2006.06.008 16904944

[DEV102186C110] Ramirez C. L., Foley J. E., Wright D. A., Müller-Lerch F., Rahman S. H., Cornu T. I., Winfrey R. J., Sander J. D., Fu F., Townsend J. A. et al. (2008). Unexpected failure rates for modular assembly of engineered zinc fingers. *Nat. Methods* 5, 374-375 10.1038/nmeth0508-374 18446154 PMC7880305

[DEV102186C111] Ran F. A., Hsu P. D., Lin C.-Y., Gootenberg J. S., Konermann S., Trevino A. E., Scott D. A., Inoue A., Matoba S., Zhang Y. et al. (2013a). Double nicking by RNA-guided CRISPR Cas9 for enhanced genome editing specificity. *Cell* 154, 1380-1389 10.1016/j.cell.2013.08.021 23992846 PMC3856256

[DEV102186C112] Ran F. A., Hsu P. D., Wright J., Agarwala V., Scott D. A. and Zhang F. (2013b). Genome engineering using the CRISPR-Cas9 system. *Nat. Protoc.* 8, 2281-2308 10.1038/nprot.2013.143 24157548 PMC3969860

[DEV102186C113] Ren X., Sun J., Housden B. E., Hu Y., Roesel C., Lin S., Liu L.-P., Yang Z., Mao D., Sun L. et al. (2013). Optimized gene editing technology for Drosophila melanogaster using germ line-specific Cas9. *Proc. Natl. Acad. Sci. USA* 110, 19012-19017 10.1073/pnas.1318481110 24191015 PMC3839733

[DEV102186C114] Reyon D., Tsai S. Q., Khayter C., Foden J. A., Sander J. D. and Joung J. K. (2012). FLASH assembly of TALENs for high-throughput genome editing. *Nat. Biotechnol.* 30, 460-465 10.1038/nbt.2170 22484455 PMC3558947

[DEV102186C115] Rong Y. S. and Golic K. G. (2000). Gene targeting by homologous recombination in Drosophila. *Science* 288, 2013-2018 10.1126/science.288.5473.2013 10856208

[DEV102186C116] Sander J. D., Dahlborg E. J., Goodwin M. J., Cade L., Zhang F., Cifuentes D., Curtin S. J., Blackburn J. S., Thibodeau-Beganny S., Qi Y. et al. (2011). Selection-free zinc-finger-nuclease engineering by context-dependent assembly (CoDA). *Nat. Methods* 8, 67-69 10.1038/nmeth.1542 21151135 PMC3018472

[DEV102186C117] Schmid-Burgk J. L., Schmidt T., Kaiser V., Höning K. and Hornung V. (2013). A ligation-independent cloning technique for high-throughput assembly of transcription activator-like effector genes. *Nat. Biotechnol.* 31, 76-81 10.1038/nbt.2460 23242165 PMC4142318

[DEV102186C118] Sebo Z. L., Lee H. B., Peng Y. and Guo Y. (2013). A simplified and efficient germline-specific CRISPR/Cas9 system for Drosophila genomic engineering. *Fly (Austin)* 8, 52-57. 24141137 10.4161/fly.26828 PMC3974895

[DEV102186C119] Shan Q., Wang Y., Chen K., Liang Z., Li J., Zhang Y., Zhang K., Liu J., Voytas D. F., Zheng X. et al (2013). Rapid and efficient gene modification in rice and Brachypodium using TALENs. *Mol. Plant* 6, 1365-1368 10.1093/mp/sss162 23288864 PMC3968307

[DEV102186C120] Smidler A. L., Terenzi O., Soichot J., Levashina E. A. and Marois E. (2013). Targeted mutagenesis in the malaria mosquito using TALE nucleases. *PLoS One* 8, e74511 10.1371/journal.pone.0074511 23977401 PMC3744473

[DEV102186C121] Sollu C., Pars K., Cornu T. I., Thibodeau-Beganny S., Maeder M. L., Joung J. K., Heilbronn R. and Cathomen T. (2010). Autonomous zinc-finger nuclease pairs for targeted chromosomal deletion. *Nucleic Acids Res.* 38, 8269-8276 10.1093/nar/gkq720 20716517 PMC3001086

[DEV102186C122] Sternberg S. H., Redding S., Jinek M., Greene E. C. and Doudna J. A. (2014). DNA interrogation by the CRISPR RNA-guided endonuclease Cas9. *Nature* 507, 62-67 10.1038/nature13011 24476820 PMC4106473

[DEV102186C123] Tan W., Carlson D. F., Lancto C. A., Garbe J. R., Webster D. A., Hackett P. B. and Fahrenkrug S. C. (2013). Efficient nonmeiotic allele introgression in livestock using custom endonucleases. *Proc. Natl. Acad. Sci. USA* 110, 16526-16531 10.1073/pnas.1310478110 24014591 PMC3799378

[DEV102186C124] Tang C., Zhang Q., Li X., Fan N., Yang Y., Quan L. and Lai L. (2014). [Targeted modification of CCR5 gene in rabbits by TALEN]. *Yi chuan=Hereditas / Zhongguo yi chuan xue hui bian ji* 36, 360-368. 24846981

[DEV102186C125] Tesson L., Usal C., Ménoret S., Leung E., Niles B. J., Remy S., Santiago Y., Vincent A. I., Meng X., Zhang L. et al. (2011). Knockout rats generated by embryo microinjection of TALENs. *Nat. Biotechnol.* 29, 695-696 10.1038/nbt.1940 21822240

[DEV102186C126] Thanisch K., Schneider K., Morbitzer R., Solovei I., Lahaye T., Bultmann S. and Leonhardt H. (2014). Targeting and tracing of specific DNA sequences with dTALEs in living cells. *Nucleic Acids Res.* 42, e38 10.1093/nar/gkt1348 24371265 PMC3973286

[DEV102186C127] Treen N., Yoshida K., Sakuma T., Sasaki H., Kawai N., Yamamoto T. and Sasakura Y. (2014). Tissue-specific and ubiquitous gene knockouts by TALEN electroporation provide new approaches to investigating gene function in Ciona. *Development* 141, 481-487 10.1242/dev.099572 24353063

[DEV102186C128] Tsai S. Q., Wyvekens N., Khayter C., Foden J. A., Thapar V., Reyon D., Goodwin M. J., Aryee M. J. and Joung J. K. (2014). Dimeric CRISPR RNA-guided FokI nucleases for highly specific genome editing. *Nat. Biotechnol.* 32, 569-576 10.1038/nbt.2908 24770325 PMC4090141

[DEV102186C129] Tsuji S., Futaki S. and Imanishi M. (2013). Creating a TALE protein with unbiased 5′-T binding. *Biochem. Biophys. Res. Commun.* 441, 262-265 10.1016/j.bbrc.2013.10.060 24148249

[DEV102186C130] Upadhyay S. K., Kumar J., Alok A. and Tuli R. (2013). RNA-guided genome editing for target gene mutations in wheat. *G3 (Bethesda)* 3, 2233-2238 10.1534/g3.113.008847 24122057 PMC3852385

[DEV102186C131] Urnov F. D., Miller J. C., Lee Y.-L., Beausejour C. M., Rock J. M., Augustus S., Jamieson A. C., Porteus M. H., Gregory P. D. and Holmes M. C. (2005). Highly efficient endogenous human gene correction using designed zinc-finger nucleases. *Nature* 435, 646-651 10.1038/nature03556 15806097

[DEV102186C132] Vasquez K. M., Marburger K., Intody Z. and Wilson J. H. (2001). Manipulating the mammalian genome by homologous recombination. *Proc. Natl. Acad. Sci. USA* 98, 8403-8410 10.1073/pnas.111009698 11459982 PMC37450

[DEV102186C133] Wang H., Yang H., Shivalila C. S., Dawlaty M. M., Cheng A. W., Zhang F. and Jaenisch R. (2013). One-step generation of mice carrying mutations in multiple genes by CRISPR/Cas-mediated genome engineering. *Cell* 153, 910-918 10.1016/j.cell.2013.04.025 23643243 PMC3969854

[DEV102186C134] Wefers B., Meyer M., Ortiz O., Hrabe de Angelis M., Hansen J., Wurst W. and Kuhn R. (2013). Direct production of mouse disease models by embryo microinjection of TALENs and oligodeoxynucleotides. *Proc. Natl. Acad. Sci. USA* 110, 3782-3787 10.1073/pnas.1218721110 23426636 PMC3593923

[DEV102186C135] Wittwer C. T. (2009). High-resolution DNA melting analysis: advancements and limitations. *Hum. Mutat.* 30, 857-859 10.1002/humu.20951 19479960

[DEV102186C136] Wood A. J., Lo T. W., Zeitler B., Pickle C. S., Ralston E. J., Lee A. H., Amora R., Miller J. C., Leung E., Meng X. et al (2011). Targeted genome editing across species using ZFNs and TALENs. *Science* 333, 307 10.1126/science.1207773 21700836 PMC3489282

[DEV102186C137] Wu Y., Gao T., Wang X., Hu Y., Hu X., Hu Z., Pang J., Li Z., Xue J., Feng M. et al. (2014). TALE nickase mediates high efficient targeted transgene integration at the human multi-copy ribosomal DNA locus. *Biochem. Biophys. Res. Commun.* 446, 261-266 10.1016/j.bbrc.2014.02.099 24589733

[DEV102186C138] Xiao A., Wang Z., Hu Y., Wu Y., Luo Z., Yang Z., Zu Y., Li W., Huang P., Tong X. et al. (2013). Chromosomal deletions and inversions mediated by TALENs and CRISPR/Cas in zebrafish. *Nucleic Acids Res.* 41, e141 10.1093/nar/gkt464 23748566 PMC3737551

[DEV102186C139] Xie K. and Yang Y. (2013). RNA-guided genome editing in plants using a CRISPR-Cas system. *Mol. Plant* 6, 1975-1983 10.1093/mp/sst119 23956122

[DEV102186C140] Yang L., Guell M., Byrne S., Yang J. L., De Los Angeles A., Mali P., Aach J., Kim-Kiselak C., Briggs A. W., Rios X. et al. (2013). Optimization of scarless human stem cell genome editing. *Nucleic Acids Res.* 41, 9049-9061 10.1093/nar/gkt555 23907390 PMC3799423

[DEV102186C141] Yoshida K., Treen N., Hozumi A., Sakuma T., Yamamoto T. and Sasakura Y. (2014). Germ cell mutations of the ascidian Ciona intestinalis with TALE nucleases. *Genesis* 52, 431-439 10.1002/dvg.22770 24619765

[DEV102186C142] Yu B., Sawyer N. A., Chiu C., Oefner P. J. and Underhill P. A. (2006). DNA mutation detection using denaturing high-performance liquid chromatography (DHPLC). *Curr. Protoc. Hum. Genet.* 48, 7.10.11-17.10.14. 10.1002/0471142905.hg0710s48 18428395

[DEV102186C143] Zhang Y., Zhang F., Li X., Baller J. A., Qi Y., Starker C. G., Bogdanove A. J. and Voytas D. F. (2013). Transcription activator-like effector nucleases enable efficient plant genome engineering. *Plant Physiol.* 161, 20-27 10.1104/pp.112.205179 23124327 PMC3532252

[DEV102186C144] Zhao P., Zhang Z., Ke H., Yue Y. and Xue D. (2014). Oligonucleotide-based targeted gene editing in C. elegans via the CRISPR/Cas9 system. *Cell Res.* 24, 247-250 10.1038/cr.2014.9 24418757 PMC3915902

[DEV102186C145] Zu Y., Tong X., Wang Z., Liu D., Pan R., Li Z., Hu Y., Luo Z., Huang P., Wu Q. et al. (2013). TALEN-mediated precise genome modification by homologous recombination in zebrafish. *Nat. Methods* 10, 329-331 10.1038/nmeth.2374 23435258

